# The Neuroprotective Effect of Methanol Extract of *Gagamjungjihwan* and Fructus Euodiae on Ischemia-Induced Neuronal and Cognitive Impairment in the Rat

**DOI:** 10.1093/ecam/nep028

**Published:** 2011-03-10

**Authors:** Bombi Lee, Eu-Jung Choi, Eun-Jung Lee, Seung-Moo Han, Dae-Hyun Hahm, Hye-Jung Lee, Insop Shim

**Affiliations:** ^1^Acupuncture and Meridian Scientific Research Center, Kyung Hee University, Seoul, Republic of Korea; ^2^Department of Internal Medicine, Won Kwang University Chonju Oriental Hospital, Chonju, Republic of Korea; ^3^Department of Biomedical Engineering, Kyung Hee University, Suwon, Republic of Korea; ^4^Department of Basic Oriental Medical Science, Acupuncture and Meridian Science Research Center, College of Oriental Medicine, Kyung Hee University, 1 Hoegi-dong, Dongdaemun-gu, Seoul 130-701, Republic of Korea

## Abstract

*Gagamjungjihwan* (GJ), a decoction consisting of five herbs including *ginseng*, *Acori Graminei Rhizoma*, *Uncariae Ramulus et Uncus*, *Polygalae Radic* and *Frustus Euodiae* (FE), has been widely used as herbal treatment for ischemia. In order to investigate the neuroprotective action of this novel prescription, we examined the influence of GJ and FE on learning and memory using the Morris water maze and studied their affects on the central cholinergic system in the hippocampus with neuronal and cognitive impairment. After middle cerebral artery occlusion was applied for 2 h, rats were administered GJ (200 mg kg^−1^, p.o.) or FE (200 mg kg^−1^, p.o.) daily for 2 weeks, followed by training and performance of the Morris water maze tasks. Rats with ischemic insults showed impaired learning and memory of the tasks. Pre-treatment with GJ and FE produced improvement in the escape latency to find the platform. Pre-treatments with GJ and FE also reduced the loss of cholinergic immunoreactivity in the hippocampus. The results demonstrated that GJ and FE have a protective effect against ischemia-induced neuronal and cognitive impairment. Our results suggest that GJ and FE might be useful in the treatment of vascular dementia.

## 1. Introduction

A variety of deficits in learning and memory function have been demonstrated in the brain of animals after injury by cerebral ischemia, such as in rats with four vessel ligation [1–3], middle cerebral artery occlusion [4, 5], permanent internal carotid artery ligation [6] and microsphere embolism [7]. Cerebral ischemia is known to produce severe histopathological damage and related behavioral deficits including cognitive and motor disorders, some of which continue to progress beyond the time of the initial insult. The middle cerebral artery-perfused brain areas such as the parietal cortex, hippocampus and striatum are mainly affected after cerebral ischemia [8, 9]. In particular, the hippocampal neurons, known to play an important role in learning and memory processes [10–12], are vulnerable to neuronal injury produced by ischemia [8, 13, 14]. Therefore, lesions of the cortex, hippocampus and striatum that lead to cerebral ischemia are well-established causes of severe deficits of learning and memory in a variety of behavioral tasks [15–18].

Cerebral ischemia has also been correlated with the degree of loss of cholinergic neurons, including the levels of acetylcholine (ACh) and choline acetyltransferase (ChAT) [3, 19]. Cholinergic neurons originating in the medial septum (MS) project to areas such as the cortex and hippocampus, which play a role in Ach-associated cognition [20, 21]. Lesions in these pathways lead to a decrease in the ACh release and cause learning and memory dysfunction, resulting from vascular dementia [22]. Many studies have suggested a relationship between learning and memory functions and the cholinergic system in experimental animals [23, 24].

In traditional oriental medicine, many herbal drugs and prescriptions have been used clinically for the treatment of stroke, Alzheimer's disease and vascular dementia. Several studies have demonstrated a variety of pharmacological actions of *ginseng* [25, 26], *Acori Graminei Rhizoma* (AGR) [27, 28], *Uncariae Ramulus et Uncus* (URE) [28, 29], *Polygalae Radix* (PGR) [30, 31] and *Frustus Euodiae* (FE) [32–34] on the central nervous system. In particular, recent studies have demonstrated that several substances present in *Gagamjungjihwan* (GJ), including ginseng, AGR and URE, have protective effects on ischemia-induced neuronal injury [28, 35].

Some studies have shown the beneficial effects of FE, the dried, unripe fruit of *Evodia rutaecarpa* belonging to the family *Rutacea* [32], and have recommended it as a treatment of headache, vomiting, colds, thoracic and abdominal pain and reduced blood circulation, as well as a treatment for stroke and vascular dementia [36, 37]. Also, many researchers have studied not only one herb or an active compound of an herb but also decoction combinations of herbs for the treatment of vascular dementia [38–40].

GJ is a decoction consisting of five herbs: ginseng, AGR, URE, PGR and FE. GJ and FE are well-known herbal medicines that have been included in medical prescriptions for a long time to treat strokes and vascular dementia. Although the prescriptions including these drugs are clinically known to be effective for stroke-induced cognitive impairment, the neuroprotective actions of GJ and FE on impairment of learning and memory in ischemia-induced cell damage have not been studied.

The purpose of the present study was to examine the effect of GJ and FE on learning and memory in ischemia-induced amnesia in rats using the Morris water maze. In addition, we evaluated the neuroprotective effects of these herbal drugs on the central acetylcholine system by assessing ChAT and AChE immunohistochemistry of hippocampal neurons.

## 2. Materials and Methods

### 2.1. Animals

Adult male Sprague-Dawley rats weighing 250–280 g were obtained from Samtaco Animal Corp. (Seoul, Korea). All animals were housed in groups of five or six with continuous access to food and water ad libitum and were maintained on a 12 h light/dark cycle regulated at 23°C room temperature. The experiments began at least 7 days after their arrival. The animal experiments were carried out in accordance with the Prevention of Cruelty to Animals Act 1986 and NIH guidance for the care and use of laboratory animals for experimental procedures, and were approved by local committee review.

### 2.2. Preparation of Methanol Extract of GJ and FE

All GJ and FE herbs were purchased from an oriental drug store (Jungdo, Inc., Seoul, Korea). The voucher specimens (No. KH-G01 for ginseng, No. KH-AGR01 for AGR, No. HP210002 for URE, No. KH-PGR01 for PGR and No. KH-FE01 for FE) are located at the herbarium located in the College of Oriental Medicine, Kyung Hee University. The GJ (ginseng 40 g, *Acori Graminei Rhizoma* 40 g, *Uncariae Ramulus et Uncus* 40 g, *Polygalae Radix* 40 g and *Fructus Euodiae* 40 g) and FE (200 g) were cut into small pieces and extracted three times in a reflux condenser for 24 h each time using 85% methanol. The solution was combined, filtered through Whatman No. 1 filter paper and concentrated using a rotary vacuum evaporator; this was followed by lyophilization. The yield of GJ and FE was 11.1 and 9.8 (w/w). The GJ and FE were then dissolved in distilled water.

### 2.3. Middle Cerebral Artery Occlusion Model

Focal cerebral ischemia was induced using the intraluminal filament technique. Anesthesia was administered with 3% isoflurane in 30% O_2_/70% N_2_O; it was maintained throughout the operation, with 0.5–0.6% isoflurane delivered via a nose mask. The right common carotid artery was exposed through a midline cervical incision. A heparinized intraluminal filament (*ϕ*0.28 mm, rounded tip) was introduced via the external carotid artery. The rectal temperature was monitored and maintained at 37°C using a heating pad (Harvard Homeothermic Blanket Control Unit, 50-7061). After 120 min of occlusion, the filament was gently pulled out and the external carotid artery was permanently closed by cauterization. In sham-operated rats, the right common carotid artery was exposed and the external carotid artery was opened without introducing the filament into the internal carotid artery. After the operation, the animals were allowed to wake up in the incubator (30°C) and were then moved to their home cages.

### 2.4. Experimental Design

Rats were divided into four groups. The experimental group was treated with GJ (200 mg kg^−1^, p.o., GJ + ISCH group (*n* = 5)) and FE (200 mg kg^−1^, p.o., FE + ISCH group (*n* = 5)) for 2 weeks after induction of ischemia. The control group was treated with saline (0.1 mg kg^−1^, p.o., SAL + ISCH group (*n* = 6)) or 2 weeks after induction of ischemia. GJ, FE and saline were administered orally everyday in the morning. The sham-operated control group (SHAM, *n* = 6) was not treated with any drug for 2 weeks after induction of sham-operated ischemia. The water maze tests were performed the third week after the induced ischemia.

### 2.5. Water Maze Task

The water maze consisted of a circular pool (painted white, 2.0 m in diameter, 0.35 m high) constructed of fiberglass. The water was maintained at a temperature of 22 ± 2°C, and was made opaque by the addition of 1 kg of powdered skim milk. During testing in the water maze, a platform 15 cm in diameter was located 1.5 cm below the water surface in one of four locations in the pool, approximately 50 cm from the sidewalls. The pool was surrounded by many cues external to the maze. A video camera was mounted in the ceiling above the pool and was connected to a video-recorder and tracking device (S-MART; Pan-Lab, Barcelona, Spain), which permitted online and off-line automated tracking of the path taken by the rat. The animals were subjected to four trials per session. The rats were trained to locate the hidden escape platform, which remained in a fixed location throughout the testing. The trials lasted a maximum of 180 s, and the latency and swim distance to find the submerged platform were recorded. The animals were tested in this way for 6 days, and then were given a probe trial on the 7th day. For the probe trials, the platform was removed from the pool and the animals were released from the quadrant opposite where the platform had been located. The length of the trial was 60 s, after which the rats were taken out of the pool. The proportion of time and swim distance the rats spent searching for the platform in the training quadrant, that is, the previous location of the platform, were recorded and used as a measure of retention. In this study, the swimming time and distance within only a 30 cm circular zone around the previous platform, that is, not in the whole quadrant, were recorded.

### 2.6. Cholineacetyltransferase (ChAT) Immunohistochemistry

At the end of the behavioral observation, the rats were anesthetized deeply with sodium pentobarbital (80 mg kg^−1^, i.p.); then they were perfused through the ascending aorta with normal saline (0.9%); this was followed by 900 ml of 4% paraformaldehyde in 0.1 M phosphate-buffered saline (PBS). The brains were removed, post-fixed overnight and cryoprotected in 20% sucrose with PBS. The brains were cut by a cryostat into 30 um coronal sections, which were processed immunohistochemically as free-floating sections. The sections were obtained according to the rat atlas of Paxinos and Watson [41], and they were stored in PBS solution for immunocytochemical processing. The sections were immunostained for ChAT by the avidin-biotin-peroxidase method. The sections were rinsed three times for 5 min each in PBS; they were then incubated for 72 h at 4°C with a primary polyclonal antiserum (rabbit anti-ChAT; Cambridge Research Biochemicals, Wilmington, DE, USA) at a titer of 1 : 2000 in PBST. The sections were washed for 5 min in PBS containing 0.3% Triton-X100 (PBST) and then incubated for 120 min in PBST containing biotinylated goat anti-rabbit IgG antibody at a 1 : 200 dilution (Vector Laboratories, Burlingame, CA, USA). Following a 90 min incubation in the Elite standard vecta stain avidin-biotin complex (ABC) reagent (Vectastain Elite Kit; Vector Lab., Burlingame, CA, USA), the sections were again washed three times for 5 min each in PBS; then they were incubated in a medium containing 0.05%  3′-diaminobenzidine tetrahydrochloride (DAB; Sigma, St. Louis, MO, USA) with 0.01% H_2_O_2_ for 1 min to reveal the immunoreactivity. Finally, the tissue was rinsed in PBS; this was followed by a brief rinse in dH_2_O, and the tissues were individually mounted onto slides. After allowing the slides to air-dry, they were coverslipped. The sections were viewed at 100× magnification and the number of ChAT-labeled cells was quantified in the hippocampus. Counts of ChAT-labeled cells were made by an observer blind to the treatment within square grids of defined size (100 *μ*m × 100 *μ*m) that were placed over each area. For measuring, the ChAT-labeled cells were counted only if they reached a defined darkness above background. Counts from the hippocampus were obtained according to the stereotaxic atlas [41]. The cells within the hippocampal areas were counted on each of three sections per each animal.

### 2.7. Acetylcholinesterase (AchE) Histochemistry

The sections were washed in PBS and incubated in a solution with 25 mg acetylthiocholine iodine for 1 h. The solution was composed of 32.5 ml of 0.1 M sodium hydrogen phosphate buffer (NaH_2_PO_4_ *·*H_2_O, pH 6.0), 2.5 ml of 0.1 M sodium citrate, 5 ml of 30 mM copper sulfate, 5 ml of 5 mM potassium ferricyanide and 5 mL of distilled water. The color of the mixing solution was a pretty green. After allowing the slides to air-dry, they were coverslipped. The sections were viewed at 100× magnification and the density of the stained nuclei of the hippocampal cells were made by an observer blind to the treatment within square grids of defined size (100 *μ*m × 100 *μ*m) that were placed over each area. The density from the hippocampal CA1 and CA3 areas were measured using Scion image program (Scion Corp., Frederick, MD, USA). The cells within the hippocampal areas were counted on each of three sections per each animal.

### 2.8. Statistical Analysis

The data were expressed as means ± SE. Group differences for the escape latency on the Morris water maze task were analyzed using a one-way analysis of variance (ANOVA) with repeated measures. One-way ANOVA followed by the Tukey post hoc test multiple group comparison was used to analyze group differences of the data collected during successive training days, probe trials, immunohistochemical assay and image analysis. A difference between groups was considered as statistically reliable if the associated probability (*P*-value) was below 0.05.

## 3. Results

### 3.1. Water Maze Test

**Figure 1:**
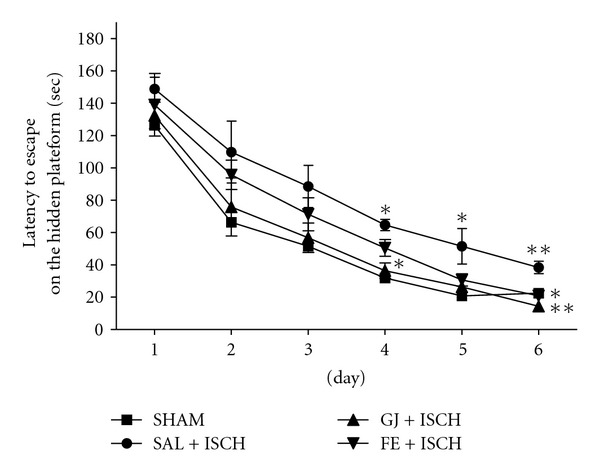
Time to escape on the platform during acquisition trials of the Morris water maze test. Four trials per day over 6 days were performed for the acquisition test. Rats were treated with GJ (200 mg kg^−1^, p.o., GJ + ISCH group, *n* = 5) and FE (200 mg kg^−1^, p.o., FE + ISCH group, *n* = 5) for 2 weeks after induction of cerebral ischemia. The sham-operated control group (SHAM, *n* = 6) and ischemia group (SAL + ISCH, *n* = 6) were not given any drug for 2 weeks after induction of ischemia. Significance with Tukey's test following an one-way ANOVA is indicated as *P <  .05, **P < .001 (Sham-operated versus SAL + ISCH, SAL + ISCH versus GJ + ISCH and SAL + ISCH versus FE + ISCH). Vertical lines indicate S.E.M (*N* = 5-6).

The forebrain ischemia affected the performance of the rats in the water maze. The SAL + ISCH group showed worse performance than the SHAM group based on significantly increased latencies for finding the hidden platform, as seen in Figure 1.

**Figure 2:**
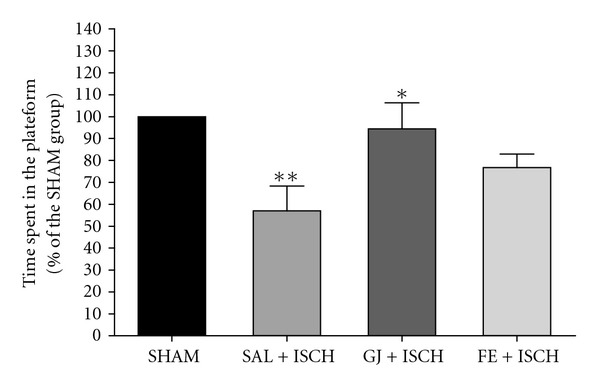
Time spent around the platform on the water maze test. The task was performed with four daily trials on the seventh day without the platform for the retention test. Rats were treated with GJ (200 mg kg^−1^, p.o., GJ + ISCH group, *n* = 5) and FE + ISCH (200 mg kg^−1^, p.o., FE + ISCH group, *n* = 5) for 2 weeks after cerebral ischemia. The sham-operated control group (SHAM, *n* = 6) and ischemia group (SAL + ISCH, *n* = 7) were not given any drug for 2 weeks after induction of ischemia. Significance with Tukey's test following an one-way ANOVA is indicated as *P < .05, **P <  .01 (Sham-operated versus SAL + ISCH, SAL + ISCH versus GJ + ISCH). Vertical lines indicate S.E.M (*N* = 5-6).

An ANOVA (4 × 6, treatment × time) performed on the swimming time in the acquisition trials revealed a significant group difference (*F*(3, 18) = 8.878, *P* < .001) and effect of day (*F*(5, 90) = 72.272, *P* <  .001]) but not a group × day interaction (*F*(15, 90) = 0.332, *P* = .990)) The Tukey's post hoc test showed that the GJ + ISCH group (*P* < .05 on days 4 and 6, resp.) and the FE + ISCH group (*P* < .05 on day 6) had significantly reduced swimming latency time, compared to the SAL + ISCH group (Figure 1). On the seventh day, the post hoc test for retention performance also showed that the GJ + ISCH (*P* < .05) group spent a longer time around the platform than the SAL + ISCH group (Figure 2).

**Figure 3:**
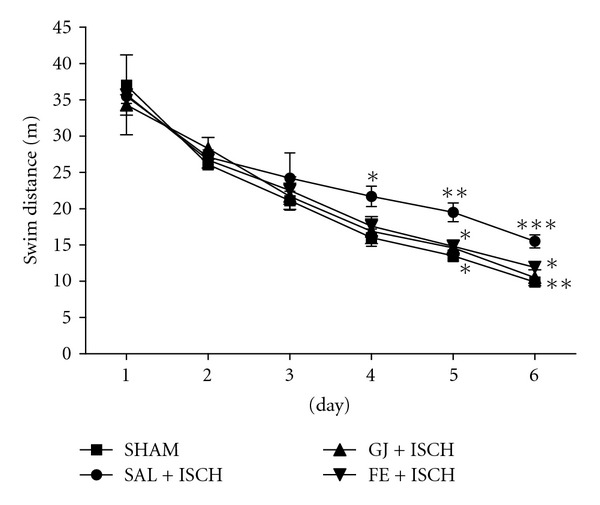
Swim distance to escape on the platform during acquisition trials of the Morris water maze test. Four trials per day over 6 days were performed for the acquisition test. Rats were treated with GJ (200 mg kg^−1^, p.o., GJ + ISCH group, *n* = 5) and FE (200 mg kg^−1^, p.o., FE + ISCH group, *n* = 5) for 2 weeks after induction of cerebral ischemia. The sham-operated control group (SHAM, *n* = 6) and ischemia group (SAL + ISCH, *n* = 6) were not given any drug for 2 weeks after induction of ischemia. Significance with Tukey's test following an one-way ANOVA is indicated as *P < .05, **P < .01, ***P < .001 (Sham-operated versus SAL + ISCH, SAL + ISCH versus GJ + ISCH and SAL + ISCH versus FE + ISCH). Vertical lines indicate SEM (*N* = 5-6).

**Figure 4:**
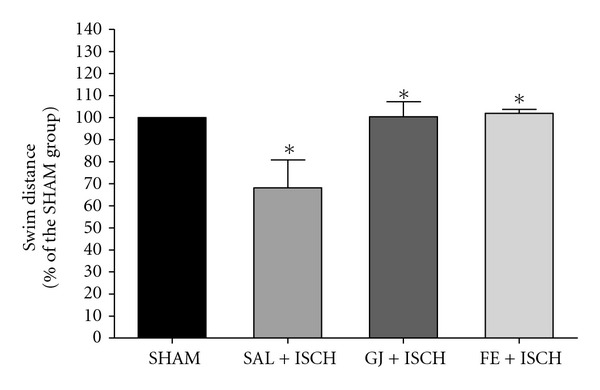
Swim distance traveled after removal of the platform on the water maze test. The task was performed with four daily trials on the seventh day without the platform for the retention test. Rats were treated with GJ (200 mg kg^−1^, p.o., GJ + ISCH group, *n* = 5) and FE + ISCH (200 mg kg^−1^, p.o., FE + ISCH group, *n* = 5) for 2 weeks after cerebral ischemia. The sham-operated control group (SHAM, *n* = 6) and ischemia group (SAL + ISCH, *n* = 7) were not given any drug for 2 weeks after induction of ischemia. Significance with Tukey's test following an one-way ANOVA is indicated as *P < .05 (Sham-operated versus SAL + ISCH, SAL + ISCH versus GJ + ISCH and SAL + ISCH versus FE + ISCH). Vertical lines indicate S.E.M (*N* = 5-6).

An ANOVA (4 × 6, treatment × time) performed on the swimming distance during the acquisition trials revealed a significant group difference (*F*(3, 18) = 4.822, *P* < .001), and an effect of day (*F*(5, 90) = 101.152, *P* < .001), but not a group × day interaction (*F*(15, 90) = 0.760, *P* = .718) (Figure 3). The Tukey's post-hoc test showed that the GJ + ISCH group (*P* < .05 and *P* < .01 on days 5 and 6, resp.) and the FE + ISCH group (*P* < .05 on days 5 and 6) demonstrated significantly reduced swimming distance compared to the SAL + ISCH group (Figure 3). On the seventh day, the post hoc test on learning and memory retention performance also revealed that the GJ + ISCH (*P* < .05) group and the FE + ISCH (*P* < .05) group spent a longer time around the platform than did the SAL + ISCH group (Figure 4). Cerebral ischemia severely impaired spatial cognition for the water maze task, but administration of GJ (200 mg) and FE (200 mg) attenuated the ischemia-induced learning and memory damage for this task.

### 3.2. Central Cholinergic System ChAT Immunohistochemistry

**Figure 5:**
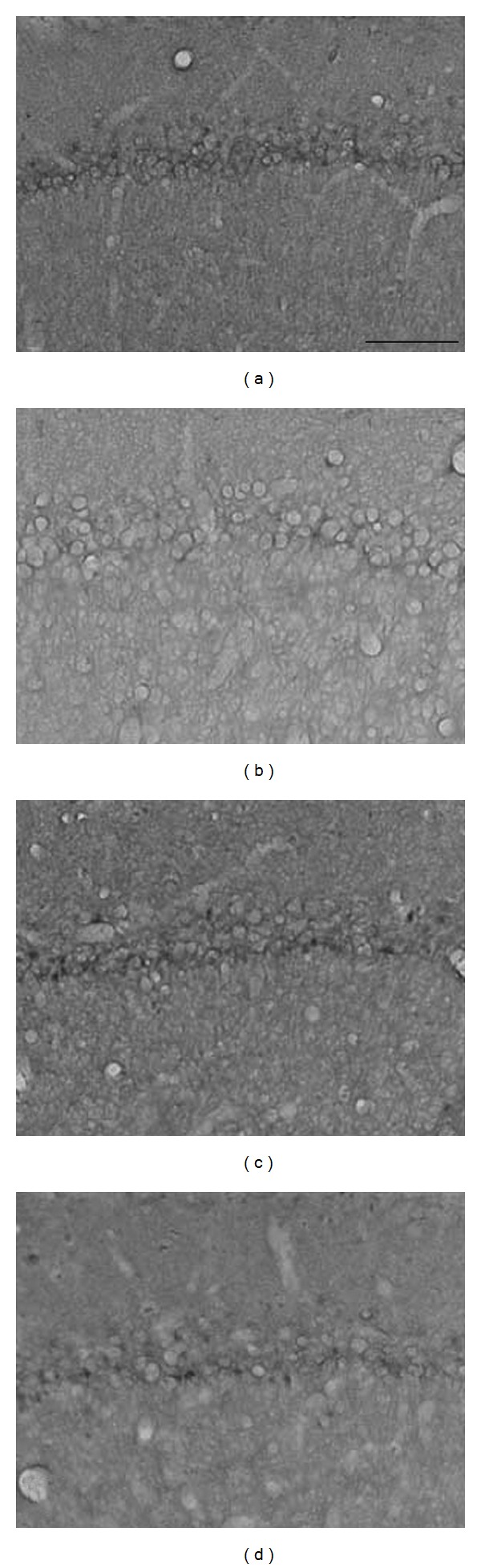
Photographs showing the distribution of ChAT immunoreactive cells in the hippocampus of SHAM (a), SAL + ISCH (b), GJ + ISCH (c) and FE + ISCH (d) groups. Rats after water maze learning task. Sections were cut coronally at 30 *μ*m and the scale bar represents 50 *μ*m (100 × 100).

**Figure 6:**
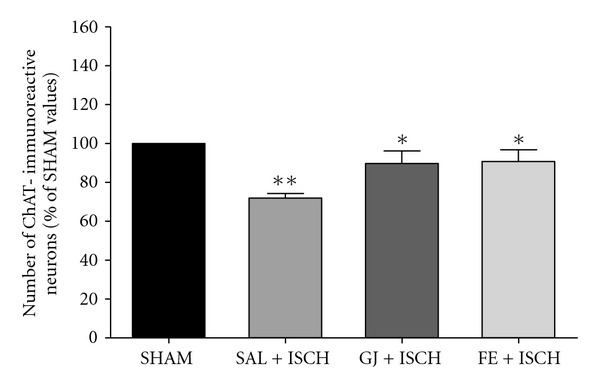
The percentage (±SE) values of quantities of choline acetyltransferase (ChAT) immunostained nuclei in different hippocampal areas of the experimental groups after the water maze learning task. Immunohistochemical data of ChAT were analyzed by performing separate one-way ANOVA of neurons among groups followed by the Tukey test. *P < .05, **P < .01, ***P < .001 (Sham versus SAL + ISCH, SAL + ISCH versus GJ + ISCH and SAL + ISCH versus FE + ISCH). Vertical lines indicate SEM (*N* = 15–18).

The results of the ChAT immunoreactivity analysis in the CA1 area are shown in Figures 5 and 6. The number of ChAT-immunoreactive neurons was 18.22 ± 1.10 (100.0 ± 0.0%) in the SHAM group, 13.11 ± 0.42 (71.95 ± 2.30%) in the SAL + ISCH group, 16.33 ± 1.19 (89.63 ± 6.55%) in the GJ + ISCH group and 16.53 ± 1.09 (90.73 ± 6.01%) in the FE + ISCH group (*F*(3, 65) = 8.373, *P* < .01) The Tukey post hoc test showed that the number of ChAT neurons significantly increased in the GJ + ISCH group (*P* < .05) and the FE + ISCH group (*P* < .05) compared to the SAL + ISCH group (in the CA1 area) (Figures 5 and 6).

### 3.3. AchE Histochemistry

**Figure 7:**
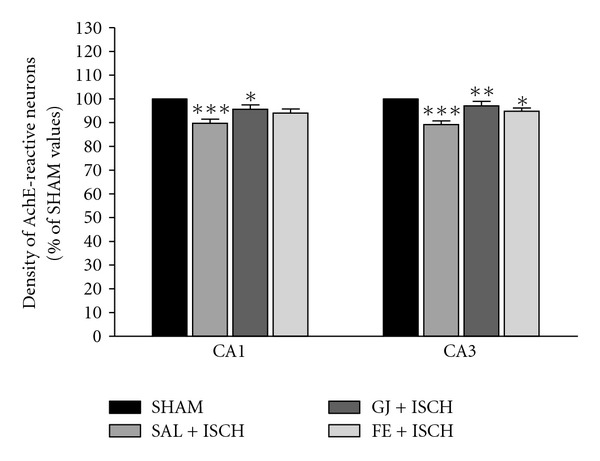
The percentage (±SE) of SHAM values of density of acetylcholinesterase (AchE) stained nuclei in different hippocampal areas of the experimental groups after the water maze learning mask. The results of AchE-reactivity were analyzed by performing separate one-way ANOVA of neurons among the groups followed by the Tukey test. *P < .05, **P < .01, ***P < .001 (Sham versus SAL + ISCH, SAL + ISCH versus GJ + ISCH and SAL + ISCH versus FE + ISCH). Vertical lines indicate SEM (*N* = 15–18).

The density of AchE fibers at the CA1 of the hippocampus was lower in the SAL + ISCH group than in the SHAM group, as shown in Figure 7. The density of the AChE neurons at the CA1 area was 123.22 ± 1.57 (100.0 ± 0.0%) in the SHAM group, 110.61 ± 2.11 (89.11 ± 1.72%) in the SAL + ISCH group, 117.87 ± 2.25 (95.65 ± 1.82%) in the GJ + ISCH group and 115.80 ± 2.20 (93.98 ± 1.78%) in the FE + ISCH group (*F*(3, 65) = 8.782, *P* < .001) The Tukey post hoc test showed that the density of the AChE reactive neurons in the hippocampus of the GJ + ISCH group (*P* < .05) was greater than that of the SAL + ISCH group (in the CA1).

The density of the AchE fibers at the CA3 of the hippocampus was lower in the SAL + ISCH group than in the SHAM group, as shown in Figure 7. The density of AChE neurons at the CA3 area was 120.44 ± 1.45 (100.0 ± 0.0%) in the SHAM group, 107.50 ± 1.76 (89.25 ± 1.46%) in the SAL + ISCH group, 116.93 ± 2.30 (97.08 ± 1.91%) in the GJ + ISCH group and 114.20 ± 1.63 (94.82 ± 1.35%) in the FE + ISCH group (*F*(3, 65) = 12.669, *P* < .001) The Tukey post hoc test showed that the density of the AChE reactive neurons in the hippocampus of the GJ + ISCH group (*P* < .05) and FE + ISCH group (*P* < .01) was greater than that of the SAL + ISCH group (in the CA3).

## 4. Discussion

The present results demonstrated that focal cerebral ischemia induced by middle cerebral artery occlusion (MCAO) produced severe deficits in performance on the Morris water maze along with signs of neurodegeneration, including decreased ChAT and AChE activity in the hippocampus. Our results showed that pre-treatment with GJ and FE attenuated the ischemia-induced learning and memory deficits on the Morris water maze and had a protective effect against ischemia-induced decrease of the cholinergic neurons.

MCAO has regional selectivity for neuronal cell death and mainly affects areas of the brain such as the hippocampus, striatum and parietal cortex [8, 9]. It has also been shown that the extent of brain damage produced by MCAO depends on the degree of the ischemic insult and its duration [19]. Therefore, consistent with a previous study [28], 2 h of exposure to MCAO, in this study, primarily produced cell death in the hippocampus, which plays a major role in learning and memory, in addition to the striatum and parietal cortex [19]. Therefore, our results suggest that the reduction of neuronal damage in the hippocampus, after MCAO, was related to the improved memory performance on the Morris water maze.

The Morris water maze is used to test relatively permanent spatial learning capabilities and reference memory; it can be used to determine whether cholinergic depletion is sufficient to produce memory impairment [11, 42]. The present study showed forebrain ischemia after MCAO, impaired behavioral performance on the Morris water maze, consistent with previous studies [15–17]. However, our findings showed that the ISCH group was not significantly different with regard to mean swimming speed, movement and rest time, an index of motor function, when compared with the sham group. Therefore, motor deficits were not demonstrated in all of animals at the time of the water maze task [43]. Our results suggest that GJ and FE improved spatial learning capability and reference memory.

In addition, the present study demonstrated that pre-treatment with GJ and FE protected the rats from loss of spatial working memory and cholinergic markers as indicated by reduction of ChAT and AChE-reactive neurons in the hippocampus, which is a particularly vulnerable region of the brain [42, 44]. We found that MCAO caused a reduction in ChAT activity in the hippocampus and significantly reduced the density of AChE in the hippocampal CA1 and CA3 regions. It is likely that the reduction in hippocampal cell loss, after treatment with the herbal drugs, was associated with the improvement of learning and memory in the rats on the water maze task. Treatment with GJ and FE produced a significant increase in the cholinergic markers, ChAT and AChE in the hippocampal pathway, compared to the ISCH group.

GJ and FE have a long history of use for ischemia therapy. Their therapeutic efficacy has been confirmed by clinical studies in the Dong-Eu-Bo-Gam (an old Korean traditional medicine book compiled by Hu Jun). Although a prescription including GJ is clinically known to be effective for stroke-induced impairment, its therapeutic effects have not been investigated. However, recent studies have shown that the five substances present in GJ have protective effects for ischemia-induced neuronal injury.

Consistent with our results, it has been reported that ginsenoside protected hippocampal neurons against ischemia [45, 46] and pre-treatment with *Panax ginseng* extract produced a cognitive enhancing effect on rats with memory impairment experimentally produced by alcohol [20]. In addition, many studies have shown that administration of ginsenosides inhibited cell death in both CA1 and CA3 regions of the rat hippocampus produced by ischemia [35, 45] and prevented the occurrence of ischemia-induced learning disability and hippocampal neuron loss in gerbils [47]. Furthermore, several studies have suggested inhibitory effects of AGR and URE on learning and memory impairments. AGR and URE protected rats from ischemia-induced neuronal death and cognitive impairment, as reported in a previous study [28]. Moreover, it has been reported that AGR has a neuroprotective effect against excitotoxic cell death and the neuroprotective effect may be through the block of NMDA receptors [48, 49]. It has been shown that a methanol extract of URE protected hippocampal CA1 cells against transient forebrain ischemia produced by a four-vessel occlusion procedure in rats [50]. PGR extract was shown to protect cultured rat granule cells against damage induced by NMDA [31], and has restorative effects on the memory and behavioral disorders produced by lesions of the nucleus basalis magnocellularis (NBM) in rats [30]. A recent study showed that methanol extract of FE protected the cardiovascular function in *N*-nitro-l-arginine methyl ester (NAME)-induced hypertensive rats and could be used for the treatment of hypertension and vascular hypertrophy [51]. The major active compound of FE, evodiamine, has been reported to exert a protective effect against myocardial ischemia-reperfusion injury in rats; this may be related to stimulation of calcitonin gene-related peptide (CGRP) release via activation of vanilloid receptors [33]. These findings suggest that dehydroevodiamine increased cerebral blood flow recorded from the surface of the supra-sylvian gyrus in anesthetized cats [34].

Therefore, these results raise the possibility that therapeutic use of crude drugs such as ARG, URE, ginseng, PGR and FE as blocking agents against the primary cause of neuronal death or memory impairment may be linked to neurodegenerative diseases.

Recently, many investigations have focused on finding a single effective compound of one herb rather than a crude herb or combinations of herbs. However, herb combinations may not only act synergistically with other constituents from the same herb but may also enhance the activity of or counteract toxic effects of compounds from other herb species [52]. Therefore, it is likely that the mechanism of action of combinations of herbs has beneficial synergistic effects. Our results may explain the differential and better effects of GJ on retention performance in the Morris water maze test than those of FE.

GJ consisted of five crude extracts of ginseng, AGR, URE and PGR including FE. These combinations contain more than 30 major active compounds such as asarone, hirsutine, onjisaponin, ginsenoside, panaxadiol and evodiamine, which are known to be effective as memory-improving therapeutic agents [53, 54]. These active compounds contained in GJ may produce better protective effects on behavioral improvement than those in FE.

One example supporting this is the new prescription of Korean red ginseng; four other herbs have been found to be more effective than the red ginseng alone for antithrombotic activity [54]. In addition, GJ, the herbal combination used in this study attenuated ischemia-induced learning and memory deficits, for the water maze in rats, and had better protective effect against ischemia-induced decrease of cholinergic neurons than did FE. The dose used, 200 mg kg^−1^ of GJ, represents approximately 40 mg kg^−1^ of each of the five herbs, 1/5–1/10 the dose used in other studies. The dosage (200 mg kg^−1^) of GJ chosen in the present study is a relatively standard dose of natural herbal medicine reported by other workers in rodent experiments and was also close to the clinically prescribed dose for human use.

Many studies have shown that ischemia-induced cell death in the hippocampal CA1 area is closely correlated with behavioral deficits in learning and memory [16], indicating that the hippocampal CA1 area plays an important role in memory processes involved in the Morris water maze. MCAO, which produced selective neuronal damage in the CA1 region of the hippocampus, causes deficit in spatial learning, as measured by an increase in escape latency and swim distance in the water maze [11, 15, 16, 55]. The analysis of our data showed a clear correlation between the reduction in hippocampal damage and the swim distance. In addition, it is likely that the reduction in hippocampal damage was due to the treatment with the herbal drugs used; this was likely the reason for the functional improvement in learning and memory. Therefore, the results showed that GJ and FE significantly improved performance on the learning tasks and prevented cell loss in the hippocampus, providing strong evidence that GJ and FE have a potential therapeutic role for the clinical treatment of cognitive impairment. However, the mechanism underlying the beneficial effects of these drugs on neural damage and cognitive impairment produced by ischemia requires further investigation.

The present study attempted to clarify the effect of GJ, composed of five neuroprotective herbs, and FE on ischemia-induced impairment of learning and memory using the Morris water maze. The neuroprotective effects of these herbal drugs on the central acetylcholine system were also examined by histochemistry of the hippocampal neurons. Pre-treatment with GJ and FE was shown to improve performance on spatial learning and memory. Moreover, treatment with GJ and FE significantly decreased the number of ChAT neurons and the density of AChE fibers in the hippocampus. The neuroprotective effects of GJ and FE are predicted by the traditional Korean medicine description of each of the five herbs.

In summary, the present results demonstrated that cerebral ischemia by MCAO produced deficits in the performance of rats on the Morris water maze and degeneration of cholinergic neurons affecting memory. Pre-treatment with GJ and FE attenuated ischemia-induced learning and memory deficits as measured by the Morris water maze and provided protective effects against ischemia-induced decrease of cholinergic neurons. Therefore, GJ and FE may be good candidates for further investigations that may ultimately result in clinical applications.

## Funding

SRC program of KOSEF (R11-2005-014); Cognitive Neuroscience Program of the Korea Ministry of Science and Technology (M10644000017-06N4400-01710), Republic of Korea.
